# Dopamine D3 Receptor Antagonists as Potential Therapeutics for the Treatment of Neurological Diseases

**DOI:** 10.3389/fnins.2016.00451

**Published:** 2016-10-05

**Authors:** Samuele Maramai, Sandra Gemma, Simone Brogi, Giuseppe Campiani, Stefania Butini, Holger Stark, Margherita Brindisi

**Affiliations:** ^1^European Research Centre for Drug Discovery and Development and Department of Biotechnology, Chemistry and Pharmacy, University of SienaSiena, Italy; ^2^Institut fuer Pharmazeutische and Medizinische Chemie, Heinrich-Heine-Universitaet DuesseldorfDuesseldorf, Germany

**Keywords:** GPCR, dopamine, drug optimization, receptor antagonists, selectivity, multi-targeting approach

## Abstract

D3 receptors represent a major focus of current drug design and development of therapeutics for dopamine-related pathological states. Their close homology with the D2 receptor subtype makes the development of D3 selective antagonists a challenging task. In this review, we explore the relevance and therapeutic utility of D3 antagonists or partial agonists endowed with multireceptor affinity profile in the field of central nervous system disorders such as schizophrenia and drug abuse. In fact, the peculiar distribution and low brain abundance of D3 receptors make them a valuable target for the development of drugs devoid of motor side effects classically elicited by D2 antagonists. Recent research efforts were devoted to the conception of chemical templates possibly endowed with a multi-target profile, especially with regards to other G-protein-coupled receptors (GPCRs). A comprehensive overview of the recent literature in the field is herein provided. In particular, the evolution of the chemical templates has been tracked, according to the growing advancements in both the structural information and the refinement of the key pharmacophoric elements. The receptor/multireceptor affinity and functional profiles for the examined compounds have been covered, together with their most significant pharmacological applications.

## Introduction

By the last midcentury, it was claimed that dopamine (DA) plays a pivotal role as neurotransmitter in the central nervous system (CNS). Due to its peculiar distribution in the brain DA controls, by interacting with its receptors, a variety of functions including locomotor activity, learning (Beninger, 1983), reward (Wise and Rompre, 1989), motivation, emotion, cognition (Nieoullon, 2002; Cools, 2008), food intake (Volkow et al., 2011), and endocrine regulation (Beaulieu and Gainetdinov, 2011). Later on, the dopaminergic system has been the focus of intense study and research, following the evidence that dysfunction of the dopaminergic system could be associated to a number of pathological conditions such as Parkinson's disease (Brooks, 2000), schizophrenia (Brisch et al., 2014), drug abuse and dependence (Volkow et al., 2007). The disclosure of specific sites of action for DA led to the identification of more than one kind of DA receptors in the brain (Sibley and Monsma, 1992). Originally, the DA receptors were classified into two subtypes, D1 and D2, owning different biochemical and pharmacological properties, and mediating distinct physiological functions. Both the D1 and D2 subtypes are G protein-coupled receptors (GPCRs) where diverse G proteins (classified in G*s*, G*i*, and G*q*) and effectors are involved in mediating their effects on different signaling pathways (Beninger, 1983).

**Figure 1:**
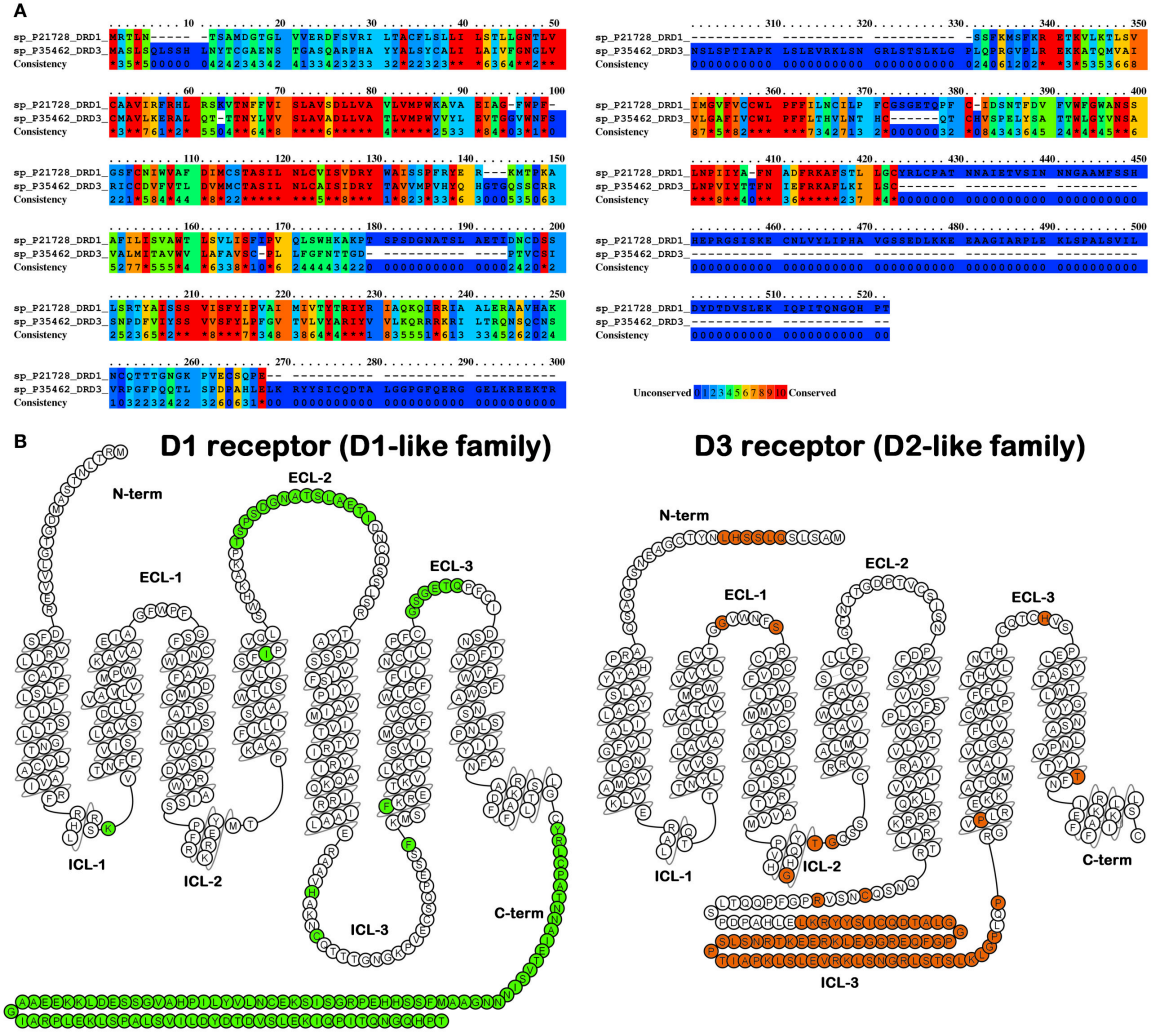
**(A)** Alignment between D1 receptor (as an example of D1-like family) and D3 receptor (as an example of D2-like family) as found by PRALINE (http://www.ibi.vu.nl/programs/pralinewww/), non-conserved residues are highlighted by dark blue background. **(B)** Snake plot representation of the above-mentioned receptors with the non-conserved residues in green for D1 receptor and in orange for D3 receptor. Plots were generated by means of GPCRdb web-server (http://gpcrdb.org/).

Subsequent biochemical studies, while suggesting the heterogeneity for the originally described D1 and D2 receptors, revealed the presence of 5 distinct receptor subtypes (Sibley and Monsma, 1992). These subtypes, based on sequence homology, were in turn clustered into two subfamilies, the so called D1-like receptors (D1 and D5) and the D2-like receptors (D2, D3, and D4). Typically, D1-like receptors are positively coupled to adenylyl cyclase (AC) and lead to intracellular cyclic 3,5-adenine monophosphate (cAMP) accumulation and activation of the protein kinase A (PKA). In contrast, D2-like receptors are negatively coupled to AC and negatively modulate the activity of PKA and its effectors (Rangel-Barajas et al., 2015). Over the past decades, a large number of agonists and antagonists for both D1-like and D2-like subfamilies have been developed and characterized (Butini et al., 2016). Although the design of ligands characterized by overall selectivity for the D1-like vs. the D2-like receptors is quite an easy task to be achieved, the development of specific ligands for a single receptor subtype has proven to be a tough challenge especially within the same receptor subfamily. Each receptor possesses an extracellular amino terminus and seven membrane spanning-helices linked by intracellular and extracellular protein loops. The carboxyl terminus is located in the intracellular space and may form a further link to the membrane. Structurally the D1-like receptors have a short third intracellular loop and a long carboxyl terminal tail, whereas the D2-like receptors display opposite features showing long third loops and short carboxyl terminus (Figure 1).

These differences provided the structural basis for the clustering of these receptors into the two mentioned subfamilies; moreover, they could also be linked to their functional outcome and significance and to specific receptor/G protein interaction potentially exploitable for the development of subtype selective ligands.

Detailed information about the structure of DA receptors, also provided by crystallographic studies for D3 subtypes (Chien et al., 2010), has greatly facilitated, in the last years, the development of subtype selective ligands.

In this review, we discuss the development of compounds behaving as D3 receptor antagonists by focusing our attention on the literature of the last 10 years. Besides a general discussion on their structure, affinity and functional profile on DA and other receptor subtypes, we also provide an overview of their application in the field of DA-related pathological states, with a particular focus on schizophrenia and drug addiction models.

## D3 receptors as members of the D2-like family of dopamine receptors

The D2-like receptor family represented by D2, D3, and D4 receptors exhibits pharmacological properties similar to those of the originally defined D2 receptor (Missale et al., 1998). Post-synaptic D2 receptors are present in dopaminergic projection areas such as the striatum (50%), limbic areas (nucleus accumbens, olfactory tubercle), hypothalamus and pituitary gland. D2 receptors are also located pre-synaptically in the substantia nigra pars compacta, ventral tegmental area and striatum, where they modulate the release of DA (De Mei et al., 2009). Activation of the striatal D2 receptor subfamily in rats results in a behavioral syndrome known as stereotypy (repetitive sniffing and gnawing, accompanied by hyperactivity). The repetitive behaviors observed in humans following amphetamine ingestion may have a similar neurochemical basis. By contrast, blockade of the striatal D2 receptor subfamily produces marked increase in muscle rigidity in rats and a Parkinson-like syndrome in humans. Administration of a D2 antagonist in humans results in a rapid and large increase in prolactin release from the anterior pituitary gland, since the physiological DA inhibition of prolactin release is blocked.

The D3 and D4 subtypes are much less abundant than the D2 subtype and have different and more restricted tissue localization. D3 receptors are predominantly located in areas considered important for psychotic symptoms such as ventral striatum including nucleus accumbens, thalamus, hippocampus, and cortex (Hall et al., 1996; Suzuki et al., 1998; Gurevich and Joyce, 1999). Some D3 receptors are also found in regions associated with motor function such as the putamen, whereas D4 receptors are found in the frontal cortex, amygdala, mid-brain and medulla.

Notably, D3 receptors possess a high affinity for DA (420-fold higher than that of D2 receptors) and, unlike D2 receptors, small changes in their number or function may lead to dramatic effects on synaptic transmission, suggesting that D3 receptors could be critical modulators of normal dopaminergic function and, despite their localization, also of cognition.

Recently, the resolution of the crystal structure of the human D3 receptor in complex with eticlopride, a potent D2/D3 antagonist (Chien et al., 2010) provided essential hints for rational drug design aiming at the development of D3 selective ligands. The crystal structure, in fact, highlighted useful structural differences between closely related GPCRs that can be exploited for drug design. In particular, the structural observation of the extracellular binding pocket, which may interact with bitopic or allosteric ligands, shed light on the role of the extracellular loops as relevant for defining a specific region for ligand binding not only at the orthosteric site (Brogi et al., 2014).

In the last decade, several evidences pointed out the dimerization phenomena of GPCRs as a pivotal issue for modulating their biological function paving the way to the future development of innovative drugs. In particular, many GPCRs have been described to form homodimers, heteromers, or oligomers (Borroto-Escuela et al., 2014). DA receptors are the most promiscuous proteins able to form dimers among the rhodopsin-like GPCRs. Dimerization can occur through their extracellular loops, transmembrane helices and intracellular loops. The dimers can be stabilized by covalent (disulphide bonds) or non-covalent (hydrophobic interactions between transmembrane helices or coiled coil structures) bonds or a combination of both. Although their physiological function is not completely understood yet, receptor dimers or oligomers have major consequences on ligand binding, activation of signaling pathways and cellular trafficking. These complexes, showing properties different from those found for each single monomer, may modify the action of DA itself and of different agonists and antagonists (Maggio et al., 2015). Therefore, targeting specific GPCR dimers may provide drugs with enhanced potency, selectivity, and therapeutic index, representing a promising alternative to conventional drug development approaches for CNS disorders.

In particular, D3 receptors can form homodimers or heteromers with D1 or D2 receptors (Agnati et al., 2016). D1-D3 receptor heterodimers have been described possessing different D3 receptor-mediated activation levels on D1 receptors, and this would make interesting to investigate the differences in the pharmacological profile of various D1 agonists. D1-D3 heteromers represent an important functional unit in the brain and are considered promising targets for neuropsychiatric disorders including Parkinson's disease and drug addiction (Guitart et al., 2014; Agnati et al., 2016). Additionally, heterodimerization was also described for D3 receptors with other GPCRs such as A2A and neurotensin receptors. Recently heteromultimers were also reported involving D3 or D2 receptors with adenosine A2A and cannabinoid CB1 receptors (Maggio et al., 2015).

## Pharmacological implications of D3 antagonism

D3 receptors have attracted interest as pharmacological targets since their peculiar anatomical distribution in the limbic areas suggests that they may play a role in cognitive and emotional functions. Accordingly, they hold a valuable potential for the treatment of neurological and psychiatric disorders being potentially devoid of the classical D2 receptor subtype side effects (Hackling and Stark, 2002; Luedtkea and Mach, 2003; Joyce and Millan, 2005; Newman et al., 2005; Kassel et al., 2015). This hypothesis has prompted many research groups to develop D3 receptor selective ligands. In this context, there is increasingly strong evidence that D3 receptor antagonists could be effective antipsychotic agents and could also be involved in behavioral sensitization, with potential efficacy in the treatment of drug abuse.

The most considerable number of reports concerns the development of D3 antagonists for the treatment of schizophrenia (Joyce and Millan, 2005) and this approach is substantiated by a series of evidences. It is well known that the inhibition of D2 receptors, which is essential for obtaining antipsychotic efficacy, is accompanied by detrimental effects on motor functions, causes extrapyramidal side effects, and increases prolactin release. In this frame, DA receptor antagonists characterized by D3 selectivity (over D2 receptors) are not expected to elicit such marked side effects (Millan et al., 2000). This lack of side effects liability is paralleled by the encouraging evidence that selective blockade of D3 receptors enhances social interaction and novel object recognition in rats (Watson et al., 2012). These rodent models efficiently mimic the negative symptoms of schizophrenia, which are poorly treated by conventional antipsychotics thus supporting D3 antagonism as a valuable approach for the treatment of this cluster of symptoms. Further, D3 receptor antagonists might also improve cognitive deficits in schizophrenic patients which are also poorly treated by currently available agents, including clozapine (Meltzer, 2004). Indeed, blockade of D2 receptors may compromise cognitive performance, while D3 antagonism was suggested to improve certain cognitive spheres. This could be due to a modulation of the cholinergic system, by increasing acetylcholine release at the prefrontal cortex level, operated by the D3 receptor antagonism (Millan et al., 2008). In fact, treatment of rats with S33138, a preferential D3 vs. D2 receptor antagonist, was demonstrated to provide enhanced efficacy against cognitive dysfunction induced by several contrasting manipulations (Millan and Brocco, 2008).

Moreover, deficits in the sensorimotor gating, assessed by the prepulse inhibition (PPI) of the startle reflex, have been reported in schizophrenia, and might correlate with the positive symptoms (Meincke et al., 2004). PPI is an operational measure of sensorimotor gating, reflected as a reduced startle response when a startling stimulus is preceded by a weaker acoustic stimulus (Zhang et al., 2007). Current data indicate that although the disruption of PPI is mediated by D2 receptors, but not D3 or D4 receptor subtypes, selective D3 antagonists can reverse the PPI-disruptive effects of other substances such as apomorphine.

The selective involvement of the D3 receptors in crucial neuronal circuits controlling motivational events triggered the identification of selective D3 receptor antagonism as a feasible therapeutic strategy against addiction (Heidbreder and Newman, 2010). This perception was further substantiated by several evidences highlighting plasticity changes in drug-addicted subjects, such as the increase in D3 receptor density in cocaine addicts and metamphetamine polydrug users (Staley and Mash, 1996; Boileau et al., 2012). Cocaine addiction represents a risk factor for schizophrenia and is likewise associated with a sensitization of the mesolimbic dopaminergic pathways and increased DA release in the mesolimbic brain area (Xi et al., 2004). In analogy with this observation, schizophrenic patients show a high incidence of abuse of drugs including cocaine.

Craving is a general central trait of addictive disorders. Drug-seeking behavior can be triggered by drug withdrawal or after a “priming dose.” Moreover, drug craving can follow the exposure to stimuli previously associated with consumption of the drug of abuse (“cue-induced craving”).

Neuroimaging studies in humans link drug-associated visual cues with DA release in the dorsal striatum and cocaine craving (Wong et al., 2006; Volkow et al., 2006, 2008). In animals, cocaine-associated cues sustain cocaine self-administration (Ito et al., 2004), increase cocaine seeking (Ciccocioppo et al., 2004), and elevate extracellular DA levels in the nucleus accumbens (Aragona et al., 2009), dorsal striatum, and amygdala (Carelli et al., 2003). A more recent study also evidences that cue-induced incubation of cocaine craving coincides with an increase of D3 (and not D1 or D2) receptor expression in the nucleus accumbens and ventral caudate-putamen in rats after prolonged withdrawal from cocaine self-administration (Conrad et al., 2010), suggesting a possible role for increased D3 receptor signaling in incubation of cocaine craving. These evidences delineate a clear direction of intervention, based on D3 receptors antagonism or partial agonism, for the treatment of craving and drug addiction.

## D3 receptor antagonists

Based on the interest that D3 receptors raised as an intriguing therapeutic target for the treatment of different neurological disorders and drug abuse, many efforts were dedicated in the last two decades to the development of D3 receptor ligands.

**Figure 2:**
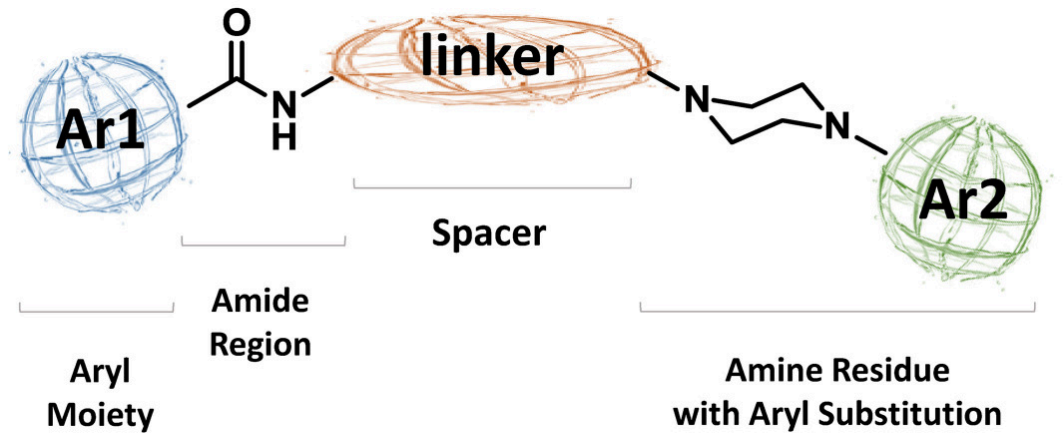
**Pharmacophore model for D3 selective ligands**.

As regards to D3 receptor antagonists, a pharmacophore model was proposed based on the structure of a series of antagonists characterized by a different degree of selectivity for D3 vs. the close homologous D2 receptor subtype (for more details see Butini et al., 2016). The model consists in an aryl moiety (Ar1 of Figure 2) linked by a H-bond acceptor function (an amide) to a spacer of appropriate length (usually four methylene units) to the basic moiety very frequently represented by an arylpiperazine system (Hackling and Stark, 2002; Löber et al., 2011).

**Figure 3:**
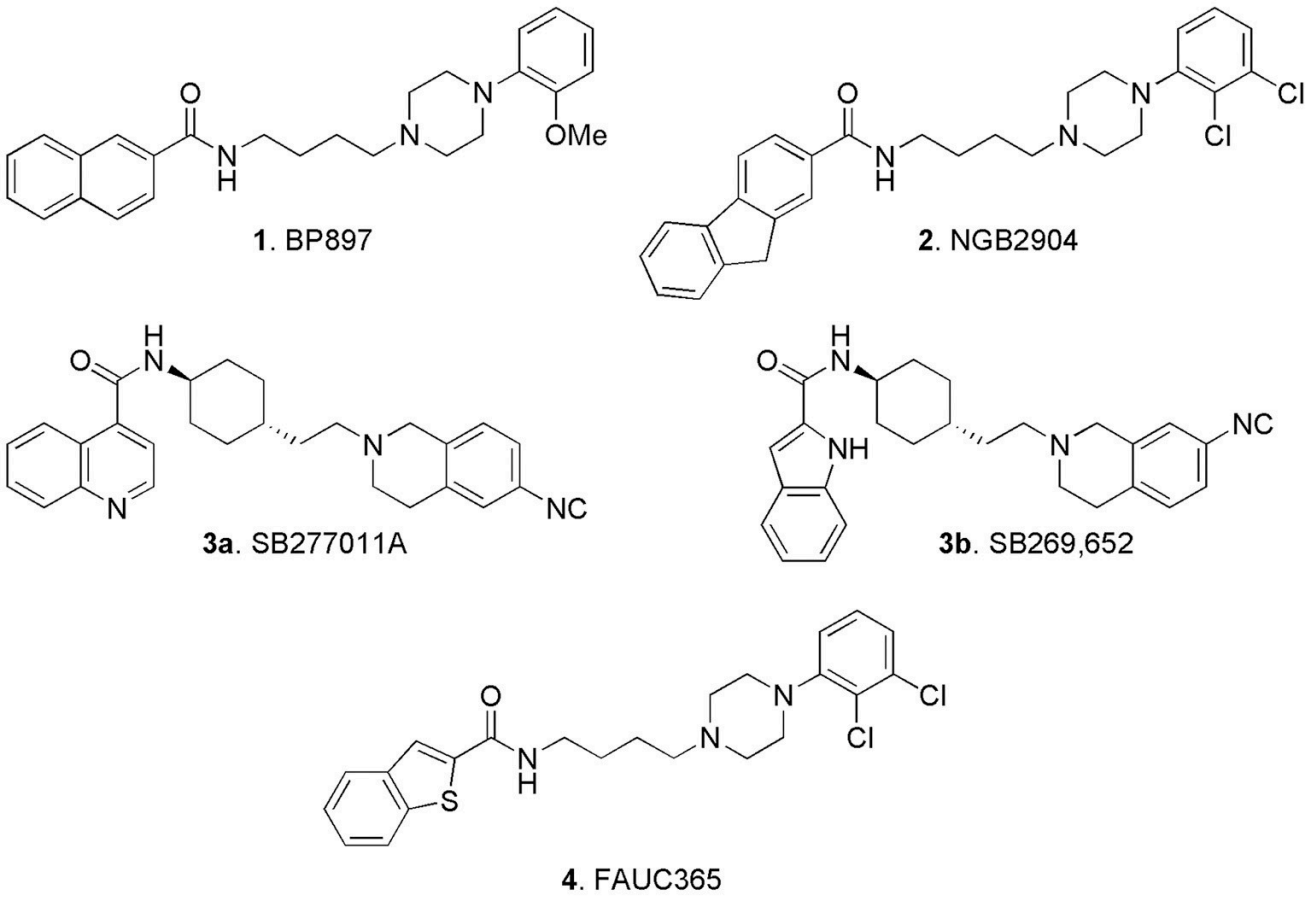
**Early identified D3 selective ligands**.

Amongst the big variety of structures reported in the literature of the last two decades, some early ligands, which were claimed as D3 receptors selective antagonists (or partial agonists) attracted particular attention and in our opinion deserve some “historical” mention: BP897 (**1**, Figure 3; Pilla et al., 1999; Garcia-Ladona and Cox, 2003), NGB2904 (**2**, Figure 3; Yuan et al., 1998; Xi and Gardner, 2007), SB277011A (**3a**, Figure 3; Reavill et al., 2000; Thanos et al., 2005), and FAUC 365 (**4**, Figure 3; Bettinetti et al., 2002).

Compound **1** was a potent DA D3 receptor ligand (*K*_i_ = 0.92 nM), showing a 70-fold selectivity vs. D2 receptors and a moderate affinity for 5-HT1A receptors, (*K*_i_ = 84 nM), adrenergic alpha1 (*K*_i_ = 60 nM), and alpha2 adrenoceptors (*K*_i_ = 83 nM). Although **1** behaved as partial agonist for the D3 receptors, it was not endowed with intrinsic activity and potently inhibited DA agonist effects in agonist-induced acidification rate or increase of GTPγS binding. This compound raised a big interest, since it was demonstrated to reduce cocaine-seeking behavior in rats (1 mg/kg i.p.) without producing reinforcement on its own, thus highlighting and endorsing the role D3 receptor for the treatment of cocaine abuse.

By substitution of the 2-methoxyphenyl- with the 2,3-dichlorophenyl- moiety linked to the piperazine nitrogen, another series of potent and selective D3 ligands was developed. The representative compound of this series, the fluorenylcarboxamide-based derivative NGB2409 (**2**), was originally described in 1998. It showed a selective D3 antagonism profile (*K*_i_ = 0.90 nM), displaying high affinity for this receptor subtype with greater than 150-fold selectivity over all other DA receptor subtypes (Yuan et al., 1998). Following studies highlighted its role in animal models of addiction, by inhibiting intravenous cocaine self-administration maintained under a progressive-ratio reinforcement schedule, cocaine- or cocaine cue–induced reinstatement of cocaine-seeking behavior, and cocaine- or other addictive drug-enhanced brain stimulation reward (Xi and Gardner, 2007).

In 2005 compound **3a** (SB277011A) was described as a brain-penetrant, high-affinity, and selective D3 receptor antagonist (p*K*_i_ = 7.95) with 100-fold selectivity over the D2 receptor and over 60 other receptors, enzymes, and ion channels (Thanos et al., 2005). Notably a structurally related compound, SB269,652 (**3b**) able to bind D2 and D3 receptors and behaving as atypical antagonist was recently reinvestigated in light of the D3 receptor dimerization. Binding kinetic studies and crystallographic analysis pointed out that **3b** behaves as dual-steric agent targeting orthosteric and allosteric binding sites of heteromers. In fact this so called “dual-steric ligand” is long enough to bridge orthosteric and allosteric binding sites, providing an exceptional selectivity for D2-D3 dimers, with relevant clinical implications (Silvano et al., 2010; Maggio et al., 2015, see also Butini et al., 2016 for further details). Structural elaboration of the D3 receptor pharmacophore allowed the identification of the benzothiophene-2-carboxamide FAUC365 **(4)** which resulted in a complete D3 receptors antagonist profile, endowed with high affinity (*K*_i_ = 0.50 nM) and selectivity (Bettinetti et al., 2002).

## Arylpiperazine-based D3 receptor inhibitors

In the following years, a number of manuscripts describing the development of arylpiperazine-based compounds appeared in the literature.

**Figure 4:**
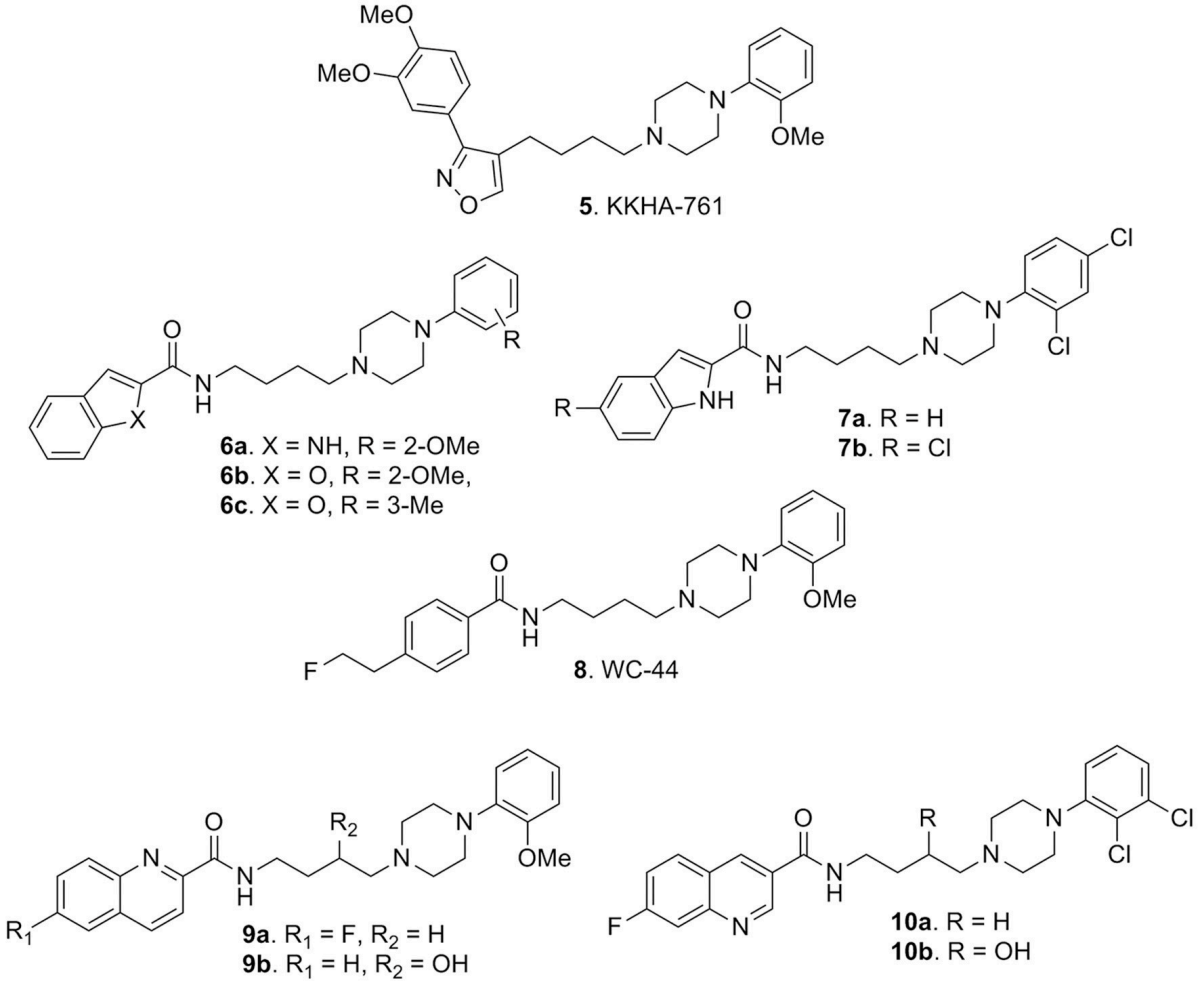
**Arylpiperazine-based derivatives 5-10**.

Amongst the variety of retrieved compounds, KKHA-761 (**5**, Figure 4) was interesting since it was described as a potent D3 receptor antagonist with high 5-HT1A receptor affinity, exhibiting antipsychotic properties in animal models of schizophrenia (Park et al., 2005). In particular, it behaved as a D2-like receptor antagonist with a high affinity for human D3 receptor (*K*_i_ = 3.85 nM) with 70-fold selectivity over the D2 receptor (*K*_i_ = 270 nM) and it also displayed high affinity for human 5-HT1A receptor (*K*_i_ = 6.4 nM). Compound **5** was characterized by a pharmacological profile tracing out that of atypical antipsychotics, like clozapine. In fact **5**, among other behavioral effects indicative of antipsychotic activity, could significantly reverse the apomorphine-induced disruption of PPI in mice thus suggesting a therapeutic potential for the treatment of anxiety, psychotic depression, and other related disorders (Park et al., 2005).

In this context, also Campiani et al. reported the synthesis of highly selective D3 receptor ligands (compounds **6a-c** and **7a,b**, Figure 4) characterized by antagonist or partial agonist activity at D3 receptors and with a D3 vs. D2 selectivity higher than 100-fold combined with 5-HT1A and 5-HT2A receptor occupancy. This represented a novel paradigm for the development of innovative and effective antipsychotics. In particular, compound **6c** emerged as interesting hit displaying high affinity for D3, 5-HT1A and 5-HT2A receptors, coupled with a low affinity for D2 receptors (to minimize extrapyramidal side liabilities), 5-HT2C receptors (to decrease the risk of obesity under chronic treatment), and for ether-a-go-go related gene (hERG) channels (to reduce cardiotoxicity). Moreover, *c-fos* expression in mesocorticolimbic areas, confirmed the atypical antipsychotic profile of **6c** *in vivo*, flanked by the absence of catalepsy at antipsychotic dose (Campiani et al., 2003; Butini et al., 2009).

In 2009 Weber and colleagues demonstrated that some derivatives such as WC-44 (**8**, Figure 4), initially identified as a selective D3 receptor agonist (*K*_i_ = 2.4 ± 0.5 nM), showed instead functional D3 receptor antagonism and possibly an innovative antipsychotic profile. Indeed, in apomorfine- and pramipexole-induced PPI deficits studies, it did not significantly oppose to PPI deficits in apomorfine experiments while it opposed to that of pramipexole-induced analysis, thus suggesting a novel antipsychotic profile for **8** linked to functional D3 receptor antagonism (Weber et al., 2009).

To the same end, Newman and co-workers prepared some 2-methoxyphenylpiperazine compounds bearing a quinoline heterocycle and explored whether the position of the quinoline nitrogen (2-, 3-, or 7-position) had any effect on binding affinity and/or selectivity. Also they inserted a 3-hydroxy substituent in the linker between the arylamide terminus and the 4-phenylpiperazine moiety. The best results, in terms of D3 affinity and selectivity, were obtained with derivatives **9a,b** (Figure 4), even though the hydroxy group at the 3-position brought a small drop of activity (*K*_i_ = 2.51 nM and 33.8 nM, respectively). The connection point of the amide functionality to the quinoline ring did not appear to strongly influence binding affinity and/or selectivity at D3 receptors, as well as the presence of an electron-withdrawing group on the quinoline ring. These compounds resulted in moderately potent antagonists, expressing weak partial agonist profiles at higher concentrations. This report further confirmed that the quinoline system represents a good scaffold for the development of D3 antagonists in terms of selectivity over D2 receptors. In fact, also derivatives **10a,b** (Figure 4), in line with their 2-methoxyphenylpiperazine counterparts **9a,b**, behaved as potent and selective D3 antagonists displaying ability in reducing heroin self-administration. The same effect was not encountered in D3 knockout mice thus clearly demonstrating the involvement of D3 receptors (Boateng et al., 2015). The data regarding intrinsic affinity described in this paper essentially match with previous findings by Campiani et al. (2003), described by the development of compounds **7a,b** which were tested in cocaine craving.

**Figure 5:**
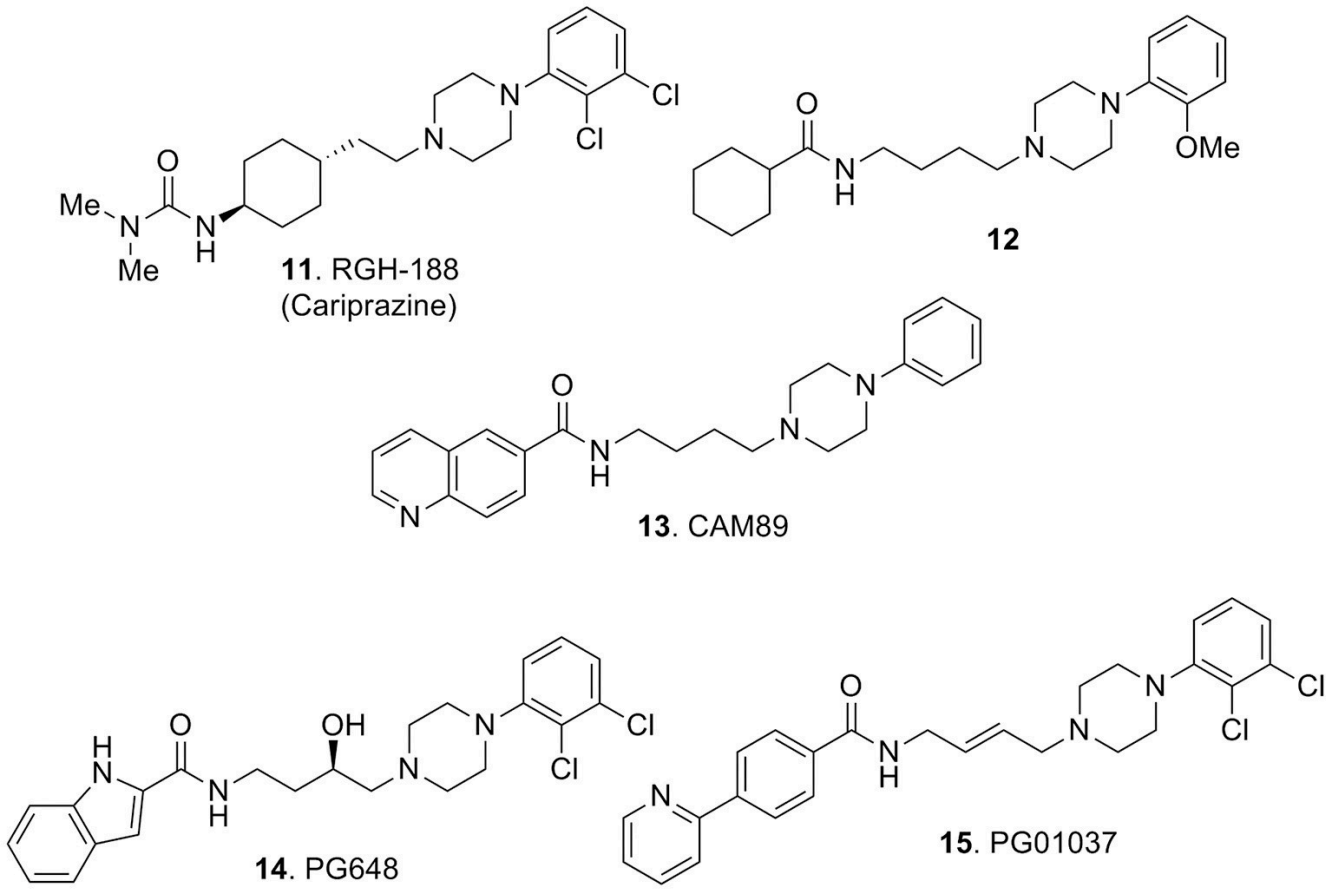
**Arylpiperazine-based derivatives 11-15**.

Substantial elaboration of the pharmacophore model of Figure 2 allowed the identification of RGH-188 (**11**, Figure 5), also known as cariprazine, a compound demonstrating subnanomolar affinity for D3 receptors and nanomolar affinity for D2 receptors (Kiss et al., 2010). This compound was developed in a medicinal chemistry approach (Agai-Csongor et al., 2012) aiming at the optimization of an impurity originally isolated during the scale-up process of a pyridylsulfonamide-based lead which behaved as D3/D2 antagonist. Cariprazine, was approved in 2015 in the USA for the treatment of schizophrenia and bipolar disorders under the trade name of Vraylar. The structural innovation that this compound brought to the pharmacophoric model of Figure 2 resided in the absence of one aromatic moiety which was efficiently replaced by a hydrophobic cyclohexyl ring system. Also the lack of the amide function connected to Ar1 of Figure 2 was replaced by an ureido moiety on the west end of the molecule. Cariprazine was demonstrated to have antagonist–partial agonist activity at both D2 and D3 receptors and animal studies highlighted its efficacy for the treatment of schizophrenia and bipolar mania (Kiss et al., 2010). Further studies demonstrated that compound **11** was able to parallel the effect of the atypical antipsychotic aripiprazole in reducing the rewarding effect of cocaine and attenuated relapse to cocaine seeking (Román et al., 2013). In adult male rats, **11** dose-dependently reversed delay-induced impairment in novel object recognition. Further, acute administration (0.1 and 0.3 mg/kg orally) to animal models of phencyclidine-induced social isolation (in neonatal rats) reduced the symptoms and induced locomotor hyperactivity (Watson et al., 2016). Seminal clinical trials allowed to confirm the therapeutic potential of **11** in patients with acute exacerbation of schizophrenia (Durgam et al., 2015, 2016). Efficacy, safety, and tolerability of **11** were assessed in patients with acute mania associated with bipolar I disorders (Calabrese et al., 2015).

Notably, some studies also evidenced the efficacy of the compound in the treatment of the negative symptoms of schizophrenia (Debelle et al., 2015). The clinical efficacy of this D3 preferring ligand further confirms the role of D3 receptors in the management of the symptoms of schizophrenia (Citrome, 2016). Learning from **11** that the aromatic group such as Ar1 of Figure 2 is not mandatory for attaining potent and selective D3 antagonism, Capet and colleagues synthesized a series of aliphatic amides and some ureidic analogs. Structure-activity relationship studies on this set of compounds showed that, as far as these products were lipophilic enough, they behaved as potent ligands (partial agonists) for human D3 receptors and the cyclohexyl amide analog **12** (Figure 5) was characterized as the best performing compound of the series (Capet et al., 2016).

One of the major drawbacks shared by many centrally active molecules, particularly those active at monoaminergic receptors, is the detection of high affinity at hERG potassium channels. Channel inhibition is associated to drug-induced torsades de pointes arrhythmia which is a major safety concern in the process of drug design and development. In fact hERG affinity is hard to be reduced in a drug design approach while retaining high receptor affinity. Unfortunately, compound **12** potently interacted with the hERG channels (82% inhibition of dofetilide binding when tested at 1 μM; Capet et al., 2016).

In a recent report describing the development of a series of arylpiperazines as potential atypical antipsychotics endowed with a multireceptor affinity profile, Brindisi and colleagues provided evidence that for compound **13** (Figure 5) they could achieve to lower hERG affinity up to 10 μM while retaining subnanomolar affinity for D3 receptors and nanomolar potency at 5-HT1A and 5-HT2A receptors [*K*_i_ (D3) = 0.6 nM, *K*_i_ (5-HT1A) = 99 nM, *K*_i_ (5-HT2A) = 66 nM; Brindisi et al., 2014]. This analog is endowed with a unique *in vitro* multireceptor pharmacological profile characterized by pronounced selectivity over the D2 (*K*_i_ (D2) > 1000 nM) and 5-HT2C receptors (*K*_i_ (5- HT2C) > 1000 nM). To further complement the significant efficacy profile of compound **13**, assessed in behavioral tests predictive of antipsychotic efficacy (e.g., MK801-induced hyperactivity and phencyclidine–induced PPI in mice) a promising therapeutic window was also ascertained by the absence of catalepsy at the antipsychotic effective dose and also in passive avoidance tests. Lack of cardiotoxicity in isolated Langendorff heart was also verified for this compound (Brindisi et al., 2014).

Newman and colleagues identified the first enantioselective D3 antagonists PG648 (*R*-**14**, Figure 5), bearing an indole system as suitable substructure for D3 ligand affinity, in which enantioselectivity was more pronounced at D3 than at D2 receptors and could represent a valuable characteristic for D3 receptor selectivity (*K*_i_ = 1.12 nM, 400-fold selective D2/D3). Interestingly, this compound also exhibited balanced physico-chemical characteristics, useful for an appropriate *in vivo* exploration and determination of intrinsic activity at D3 receptors in animal models of addiction and other neuropsychiatric disorders (Newman et al., 2009).

The 2-pyridylphenyl analog **15** (Figure 5) with a *K*_i_ = 0.7 nM for D3 receptors and a D2/D3 selectivity ratio of 133, originally described in 2005 and after evaluation in animal models of cocaine abuse, served as an important pharmacological tool for highlighting the contribution of D3 receptors in drug reinforcement *in vivo* (Grundt et al., 2005). A more recent investigation of the same compound reported its useful application on methamphetamine self-administration, methamphetamine-associated cue-induced reinstatement of drug seeking and methamphetamine-enhanced brain stimulation reward, thus highlighting the possible role of D3 antagonists also in the treatment of methamphetamine addiction (Higley et al., 2011).

**Figure 6:**
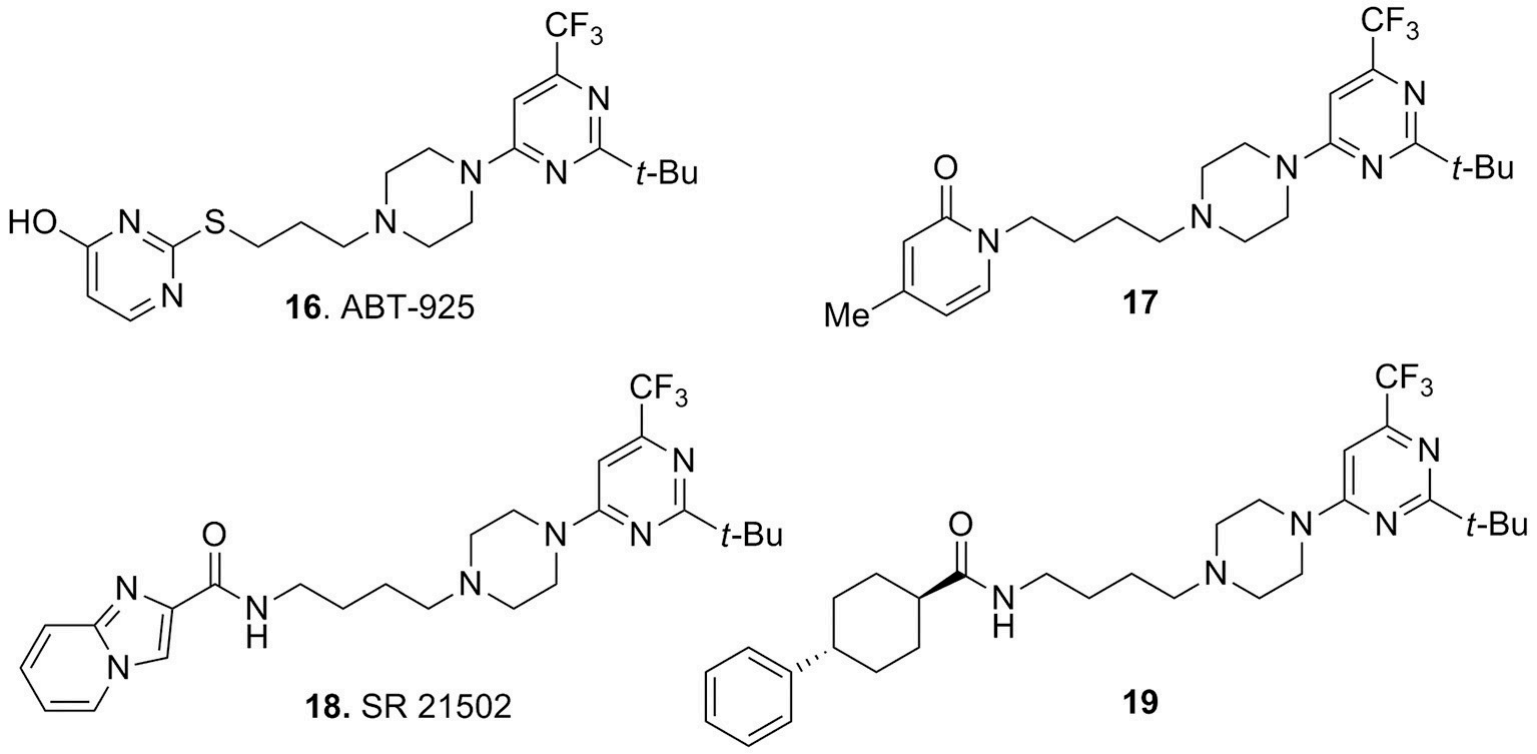
**Pyrimidinylpiperazine-based compounds 16–19**.

A series of interesting compounds displayed a *tert*-butyl-trifluoromethylpyrimidine moiety connected to the piperazine. An early lead of this series was compound ABT-925 (**16**, Figure 6), which showed high affinity for the human D3 receptor (*K*_i_ = 2.9 nM), with at least 100-fold selectivity over the human D2 and other receptors, enzymes, and ion channels. This compound was a potent antagonist of D3 receptors, it could easily cross the blood–brain barrier and exerted efficacy in different animal models predictive of antipsychotic activity without inducing catalepsy or raising plasma prolactin levels (Geneste et al., 2006). The same report also described the methylpyridin-2-one analog **17** (Figure 6) which retained a good activity and selectivity for D3 vs. D2 receptors (*K*_i_ = 0.8 nM, with >80-fold selectivity vs. D2 receptors). However, within this class of compounds the best performances were achieved with the disclosure of SR21502 (**18**, Figure 6) which displayed a *K*_i_ = 4.2 nM at the D3 receptors with >120-fold of selectivity over the D2 receptors. Although, **18** behaved as weak partial agonist at the D3 receptors, in the agonist-stimulated mitogenesis assay, it behaved as an antagonist at D3 receptors (Ananthan et al., 2014). Compound **18** was recently employed as a pharmacological tool for evaluating its *in vivo* activity against cocaine reward and cocaine-seeking behaviors. Experimental data demonstrated efficacy of **18** against these behaviors which was not accompanied by effect on food reward or spontaneous locomotor activity (Galaj et al., 2014; Hachimine et al., 2014).

Among the pyrimidinylpiperazine derivatives, compound **19** (Figure 6) bearing a phenylcyclohexanecarboxamide moiety was recently characterized as a potent D3 vs. D2 receptors antagonist (*K*_i_ = 9.4 nM) with >150-fold selectivity over D2 receptors, thus fostering further investigation for these analogs as antipsychotics or in models of drug addiction (Ananthan et al., 2014).

**Figure 7:**
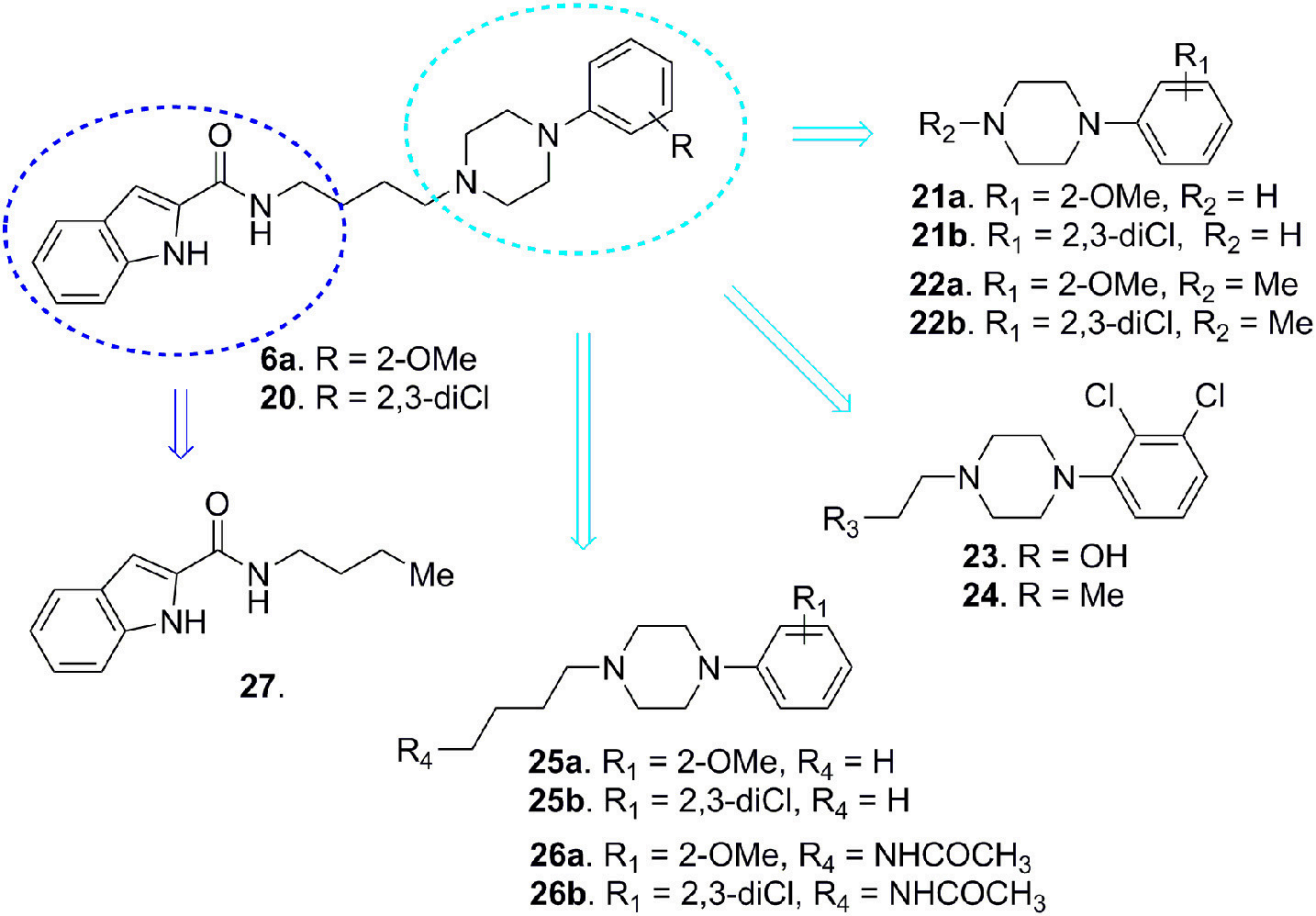
**Deconstruction studies performed on D3 receptor ligands 6a and 20**.

In order to elucidate the structural features responsible both for D3 vs. D2 receptors efficacy and selectivity, in 2012 Newman and co-workers reported a deconstruction study on substituted-4-phenylpiperazines **6a** and **20** (Figure 7; Newman et al., 2012).

**Figure 8:**
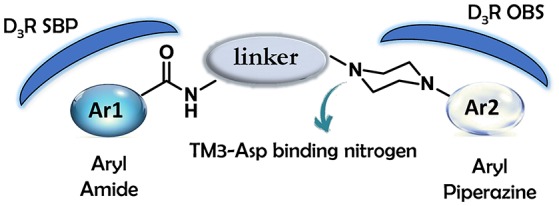
**Schematic representation of the flexible arylpiperazinecarboxamides binding the OBS and SBP of the D3 receptor**.

D3 receptors feature an orthosteric binding site (OBS), which binds DA, that is very similar to that of D2 receptors, and this represents a major issue in the development of D3 selective ligands. The Authors evidenced how selectivity could be managed by means of divergent interactions within a second binding pocket (SBP) which is distinct from the OBS. Notably, they also indicated that, the binding mode at SBP is highly influenced by that observed at the OBS. For reaching this conclusion the Authors studied the binding mode (by computational analysis) and affinity profile of a series of fragments (**21-27**, Figure 7) of compounds **6a** (Campiani et al., 2003) and **20** (Chu et al., 2005). They identified a primary pharmacophore element (the arylpiperazine moiety, Figure 8) which should bind the OBS, and a secondary pharmacophore element (the arylamide moiety, Figure 8) which should bind the SBP. Interestingly, these aspects could be translated to other GPCRs and, as a general “rule,” it could be assumed that the SBP can be targeted by bitopic or allosteric ligands.

**Figure 9:**
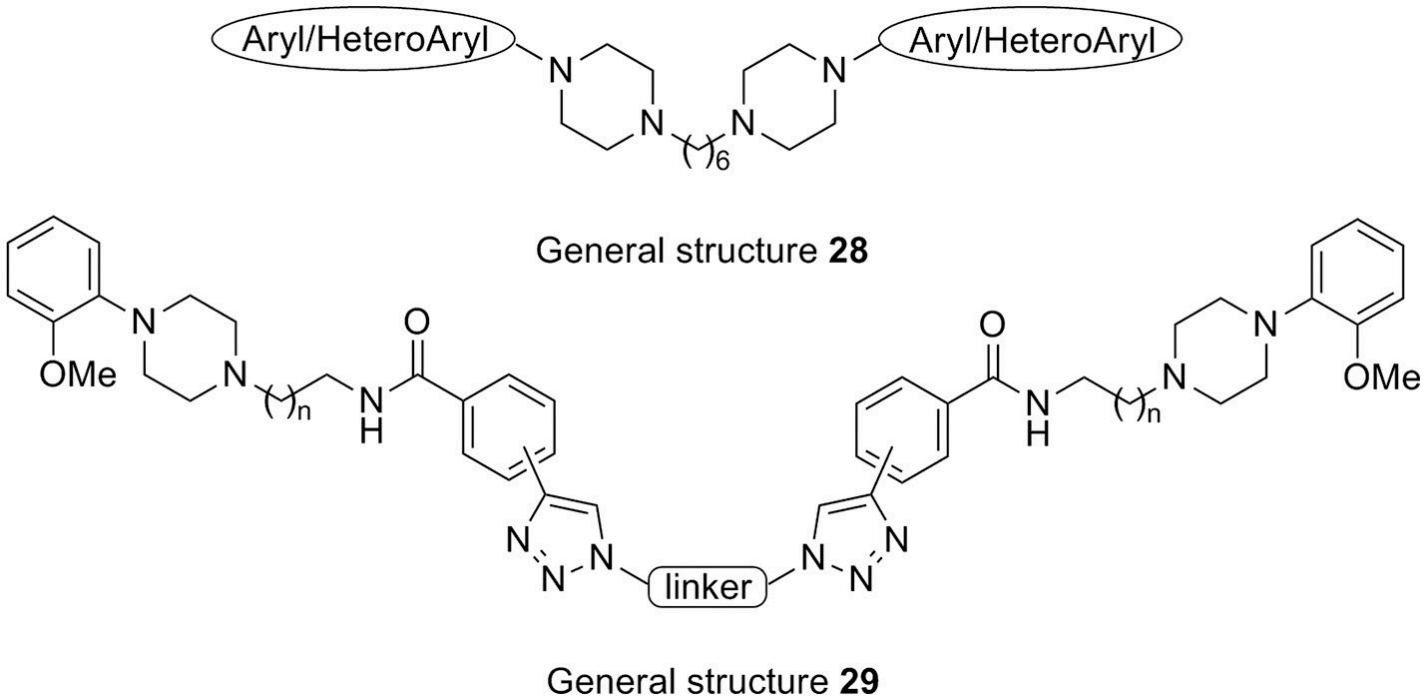
**Arylpiperazine-based D3 “dimeric” and bitopic ligands 28 and 29**.

An earlier report by Butini and co-workers described the development of bishomo- or hetero-arylpiperazines (**28**, Figure 9) as flexible ligands for specific occupancy of D3, 5-HT1A, and 5-HT2A receptors. These compounds represent a different class of ligands when compared to the general structure of the proposed pharmacophoric model (see Figure 2; Butini et al., 2010). However, the same compounds may also comply with the above mentioned study of Newman and coworkers, but the nature of these homodimeric or heterodimeric ligands implies that both the primary pharmacophore element and the secondary pharmacophore element are arylpiperazines indeed. The same consideration may be true for bitopic ligands such as **29** (Figure 9) developed by Huber and colleagues in 2009 (Huber et al., 2009).

**Figure 10:**
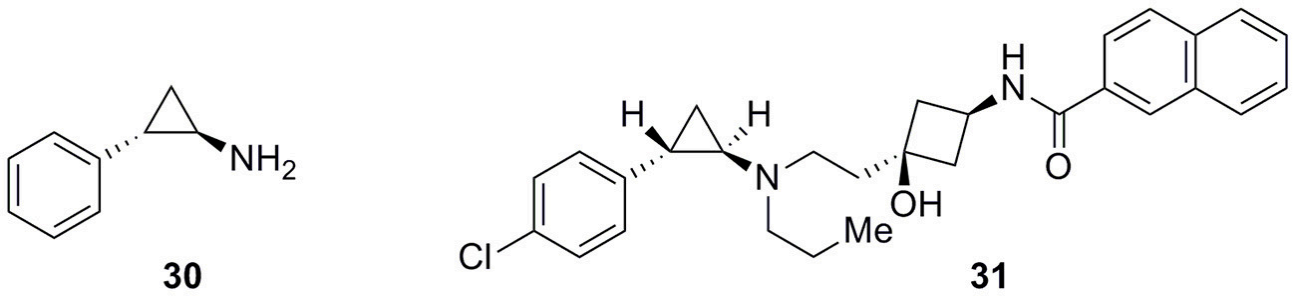
**Tranylcypromine (30) and Tranylcypromine-based analog 31**.

A further structural modification characterizes the new compound **31** (Figure 10), developed by Chen and co-workers, with marked differences from the classical arylpiperazine selective ligands. However, **31** still perfectly fitted the arlypiperazine–based pharmacophore of Figure 2. Compound **31** was a potent and selective D3 receptor antagonist based upon tranylcypromine (**30**, Figure 10) which effectively replaced the arylpiperazine moiety (Chen et al., 2014).

Although **30** had a low affinity for rat D3 receptors (*K*_i_ = 12.8 μM), derivative **31** showed *K*_i_ values of 2.7 and 2.8 nM at the rat and human D3 receptors, respectively, and displayed a high selectivity over the rat and human D2 receptors (>10,000-fold and 223-fold respectively). These features of compound **31** complemented by a good pharmacokinetic profile and brain permeability made it a promising candidate for the potential treatment of drug abuse.

## 1,2,4-triazole-based D3 receptor ligands

The arylpiperazine system proved to be an exceptionally valuable option for the design and development of selective D3 ligands, endowing the new compounds with different *in vitro* outcomes as both partial agonists and antagonists. However, in the last years, alternative scaffolds emerged such as 1,2,4-triazole-based derivatives which have been characterized as optimal alternative to the more conventional arylpiperazine-based pharmacophore of Figure 2. These studies led to the discovery of interesting structures potentially useful for the treatment of schizophrenia and related disorders.

**Figure 11:**
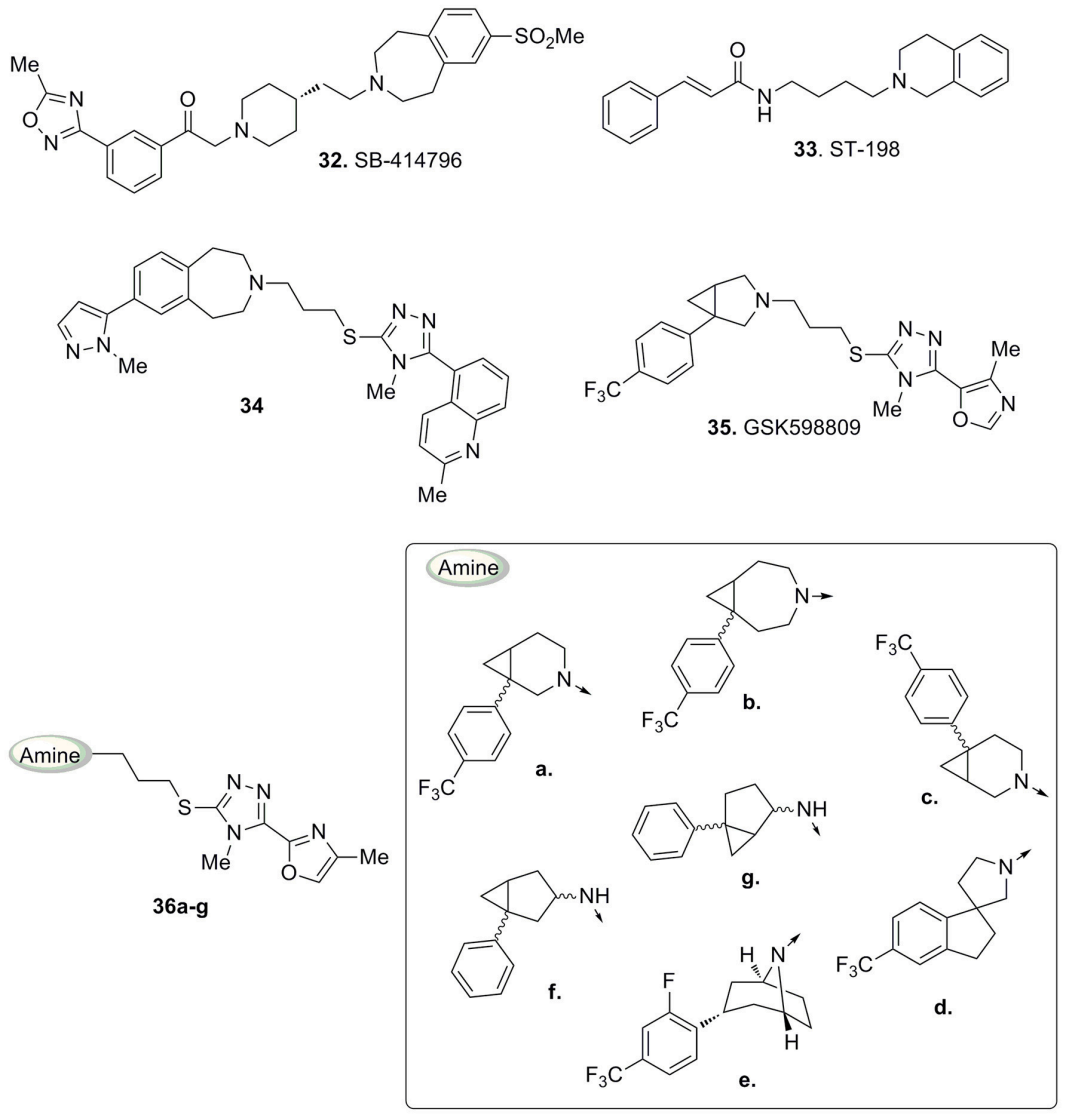
**Compounds 32 and 33 and structurally related triazole-based D3 antagonists 34–36**.

An early series of triazole-based D3 antagonists was developed by drawing inspiration from the structure of compound **32** (Figure 11), a tetrahydro-1*H*-3-benzazepine which was described as a potent and selective D3 receptors antagonist with high oral bioavailability and blood-brain barrier permeability (Macdonald et al., 2003).

Also compound ST-198 (**33**, Figure 11), developed by Bézard and colleagues, bearing a tetrahydroisoquinoline moiety, resulted in a selective D3 antagonist over a wide panel of receptors (Bézard et al., 2003).

Subsequently, with the development of compound **34** (Figure 11) Micheli and co-workers highlighted that the thiotriazole scaffold coupled to a pyrazolyl moiety represented a promising combination for the design of D3 antagonists. In particular, **34** (Figure 11) showed good oral bioavailability and brain penetration associated with high potency and selectivity for D3 receptors *in vitro* (functional p*K*_i_ obtained from the GTPγS functional assay = 8.8). An in depth *in vivo* characterization of **34** showed its ability to prevent nicotine-induced conditioned place preference behavior in rats and to reduce alcohol self-administration. Moreover, it retained a low interaction with hERG channels and no QTc interval prolongation was observed in electrocardiograms, thus indicating a favorable potential for **34** to turn into an optimal candidate for the treatment of drug addiction, psychosis, and schizophrenia (Micheli et al., 2007).

**Figure 12:**
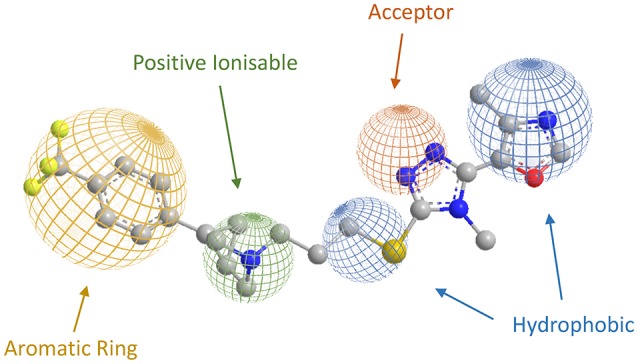
**Representation of the novel pharmacophoric model proposed by Micheli et al. (Bonanomi et al., 2010; Micheli et al., 2010b), with compound 35 and its features**.

More recently Micheli and collaborators reported an ample series of 1,2,4-triazolyl azabicyclo[3.1.0]hexanes as selective D3 receptors antagonists in which the oxazolyl derivative **35** (Figure 11) showed good affinity and selectivity coupled with optimal pharmacokinetic properties (Bonanomi et al., 2010; Micheli et al., 2010b). Successively, compound **35** was used in studies regarding food cues in a human addict population, where it showed a lack of attentional bias toward food if compared with the placebo (Nathan et al., 2012). These data provided additional support that antagonism for D_3_ receptors may attenuate attentional processing of salient or rewarding cues. On the basis of the abovementioned results, the same Authors proposed a new pharmacophore model useful for the rationalization of the activity and selectivity profiles of this new series of compounds (Figure 12).

This also allowed the identification of alternative scaffolds to the azabicyclo[3.1.0]hexane, by the analysis of differently decorated amines that nicely fitted in the proposed pharmacophore model and prompted the synthesis of derivatives (**36a-g**, Figure 11; Micheli et al., 2010a).

**Figure 13:**
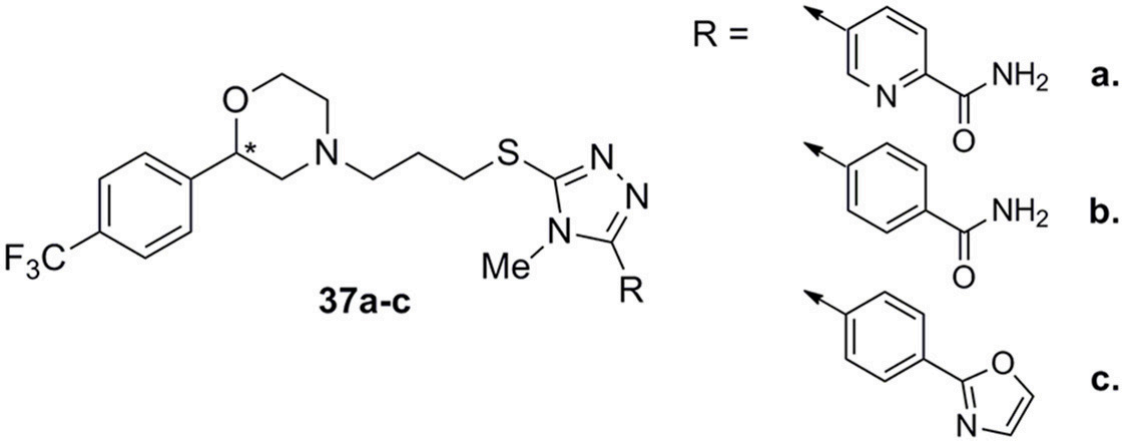
**Morpholino/triazole analogs 37a–c**.

Very recently, Micheli and colleagues reported the development of D3 ligands by means of a “scaffold hopping” strategy that resulted in the identification of a variety of original basic moieties and, more specifically, a morpholine system, to be spaced from the thiotriazole by a three-methylene tether (Micheli et al., 2016b). Derivative **37a** (Figure 13), one of the most active and selective D3 ligand of this interesting series, displayed a very high D3/D2 selectivity (800-fold) accompanied by a 60-fold selectivity vs. the hERG channel. Structure-activity relationship studies demonstrated that shift of the position of pyridine nitrogen reduced the affinity at D3 receptors, while its removal (benzamide **37b**, Figure 13) still allowed to maintain high D3 receptors affinity and D3/D2 selectivity accompanied by a notable 150-fold selectivity vs. the hERG channel. The compound bearing the oxazole moiety (**37c**, Figure 13) surprisingly showed increased affinity at the D3 receptors with 300-fold selectivity vs. D2 and 200-fold vs. hERG channel. These compounds, bearing a chiral center at the morpholino system level, were described and tested as racemates, although some of the pure enantiomers were tested showing different selectivity profile, highlighting a stereoselective interaction with DA receptors.

**Figure 14:**
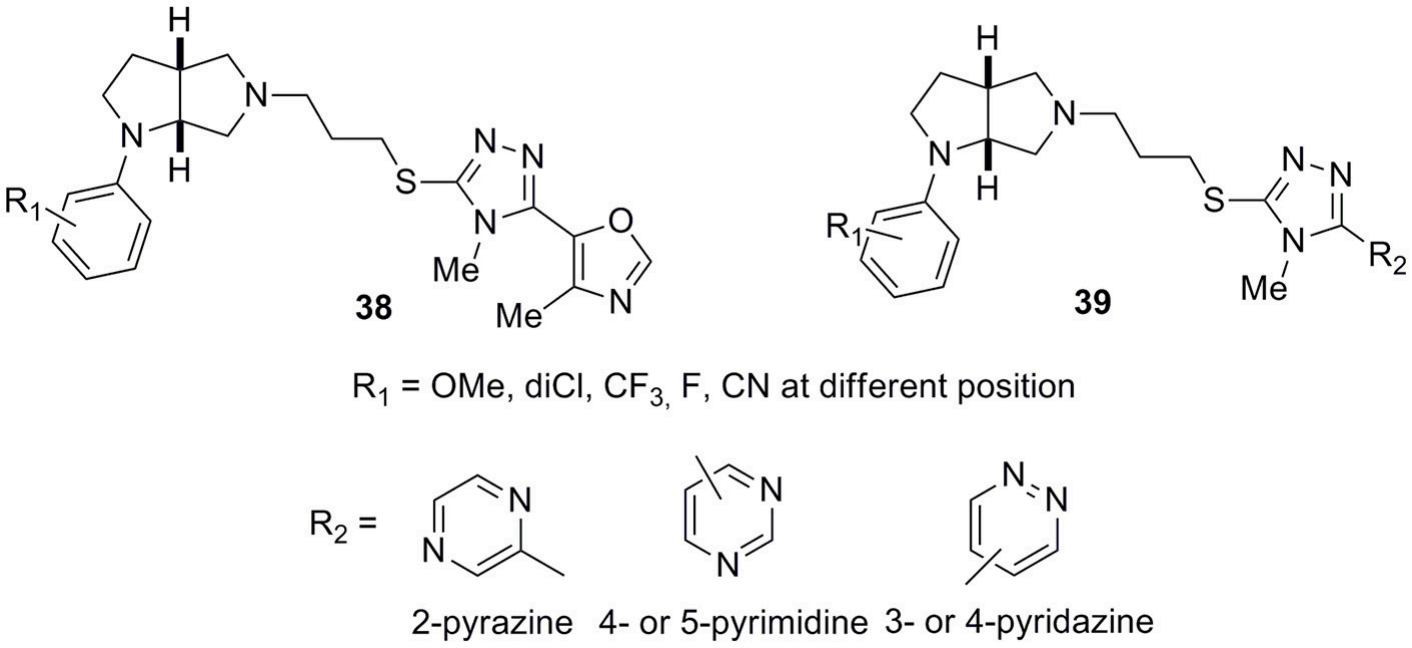
**General structure of octahydropyrrolo[2,3-***b***]pyrrole-based D3 antagonists 38 and 39**.

Another interesting and very wide series of ligands was reported by the same Authors (Micheli et al., 2016a) bearing an octahydropyrrolo[2,3-*b*]pyrrole scaffold characterized by high affinity and selectivity at D3 receptors (general structures **38** and **39**, Figure 14). Substituents at R1 position were -OMe, 2,3-dichloro, -CF_3_ groups or fluorine atoms in general structure **38**, while R2, when different from the oxazole ring, could be a pyridine, pyrazine, pyrimidine, or pyridazine moiety, combined with the above listed R1 substituents (general structure **39**). Many of these derivatives proved to have ideal *in vitro* pharmacokinetic developability and, for some selected analogs, a large selectivity panel assessment campaign was performed including hERG channels.

## Conclusion

In this review, an overview of the literature of the last 10 years in the field of D3 antagonists was provided. The careful examination and clustering of the most significant compounds taken into consideration allowed delivering an overall picture of their structural variability. At the same time, the proposed analysis highlighted the substantial structural commonalities, despite the efforts of different research groups for providing pharmacophore refinements aiming at the discovery of innovative scaffolds for attaining high D3 receptor affinity and selectivity over the D2 receptors. The description of the pharmacodynamic (multi)receptor affinity profile and the most significant pharmacological applications for the examined compounds in the field of DA-related brain disorders were also covered, in order to provide clear-cut hints for their therapeutic potential.

## Author contributions

SM wrote the paper. SG collected and clustered the literature data. SiBr collaborated in writing the introduction section and prepared the figures. GC collaborated in literature selection with particular reference to D3 antagonists as antipsychotics. SB collaborated to paper writing, supervised the overall work and revised the paper. HS collaborated in the reference literature selection and revised the paper. MB wrote the paper.

### Conflict of interest statement

The authors declare that the research was conducted in the absence of any commercial or financial relationships that could be construed as a potential conflict of interest.
